# The impact of arterial flow complexity on flow diverter outcomes in aneurysms

**DOI:** 10.1038/s41598-020-67218-9

**Published:** 2020-06-25

**Authors:** Kamil Jerzy Chodzyǹski, Pierrick Uzureau, Vincent Nuyens, Alexandre Rousseau, Gregory Coussement, Karim Zouaoui Boudjeltia

**Affiliations:** 1 Laboratoire de Médecine Expérimentale (ULB222), CHU Charleroi, Université Libre de Bruxelles, 6110 Montigny le Tilleul, Belgium; 2 Department of Fluids-Machines, University of Mons, 7000 Mons, Belgium

**Keywords:** Aneurysm, Blood flow

## Abstract

The flow diverter is becoming a standard device for treating cerebral aneurysms. The aim of this *in vitro* study was to evaluate the impact of flow complexity on the effectiveness of flow diverter stents in a cerebral aneurysm model. The flow pattern of a carotid artery was decomposed into harmonics to generate four flow patterns with different pulsatility indexes ranging from 0.72 to 1.44. The effect of flow diverters on the aneurysm was investigated by injecting red dye or erythrocytes as markers. The recorded images were postprocessed to evaluate the maximum filling of the aneurysm cavity and the washout time. There were significant differences in the cut-off flows between the markers, linked to the flow complexity. Increasing the pulsatility index altered the performance of the flow diverter. The red dye was more sensitive to changes in flow than the red blood cell markers. The flow cut-off depended on the diverter design and the diverter deployment step was crucial for reproducibility of the results. These results strongly suggest that flow complexity should be considered when selecting a flow diverter.

## Introduction

Flow diverters are common devices for treating large or complex intracranial aneurysms. As the mechanisms underlying aneurysm occlusion are still not fully understood, the choice of flow diverter is mainly empirical and based on the physician’s own experience^1,2^. Outcomes range from complete or partial occlusion after varying periods to lack of thrombosis or even haemorrhage^3,4^. The factors responsible for occlusion following flow diverter placement are still under debate^3,5^. Analysis of the flow inside large aneurysms reveals that, by contrast with small aneurysms where flow is similar to the parent vessel, the flow pattern can be markedly altered^6^. Recent studies show that flow complexity is a key factor in flow diverter performance. Computational fluid dynamics (CFD) simulations showed that flow reduction following flow diverter treatment was more noticeable during diastole than systole and that flow diverter performance was related to the flow pattern^7,8^. Although blood flow complexity can be fully described using a Fourier decomposition into harmonics, these values are not commonly used in the medical field^9^. Instead, the pulsatility index, which can be directly assessed from Doppler or magnetic resonance imaging (MRI) examinations, is more widely used to describe the complexity of patient blood flows^10^. The pulsatility index varies within the cerebral arterial tree and within the same artery in different patients, reflecting the diversity of flow patterns that can be encountered within the vessels altered by aneurysms^11,12^. Different outcomes are therefore observed when the same flow diverter is used in situations of different flow patterns associated with vessel geometry and position in the cerebral arterial tree, and the physiological state and activity of the patient^13,14^. In addition, flow diverter design, including the number of layers, porosity, number of holes, weave angle, wire diameter, and weave pattern, can affect the flow alteration induced by the flow diverter^7,13^. The relationship between flow diverter properties and flow patterns is under intense scrutiny^3^.

Researchers and physicians have used different approaches to assess flow diverter capabilities. Current data come essentially from patient follow-up, experimental validation and mathematical modeling^4,7,15,16^. Although clinical studies provide data about ability to cause aneurysm occlusion, these results are patient specific and difficult to generalise^3,4,17^. By contrast, numerical simulations are highly flexible and can evaluate the outcomes of any flow diverter–patient configurations but are limited by the complexity of the algorithms and the consequent computation cost to obtain realistic results^7,13,14^. The complexity of the blood flow in the aneurysm is one of the major pitfalls of such an approach. Experimental approaches rely on *in vivo* or *ex vivo* animal studies and on *in vitro* silicone models of altered vessels using patient-specific blood flow conditions^15,16,18^. While experimental models are limited due to species differences, silicone models rarely test liquid media that accurately mimic blood flow complexity.

In this article, the impact of harmonic flow complexity on flow diverter treatment of intracranial aneurysms was investigated experimentally using an *in vitro* analysis. For this purpose, a silicone model of a spherical aneurysm was connected to a test bench mimicking the pulsatile haemodynamic conditions encountered *in vivo*^19^. For the input flow signal, the flow from a reference patient was decomposed into four patterns with decreasing complexity and pulsatility indexes. To monitor the flow behaviour inside the aneurysm, we injected red dye or red blood cells (RBCs) as a marker into the circuit. The *in vitro* experiments were conducted with and without 6 types of flow diverter, presenting distinct porosities, numbers of holes, and weave angles.

## Results

### Selection of flow patterns

**Figure 1:**
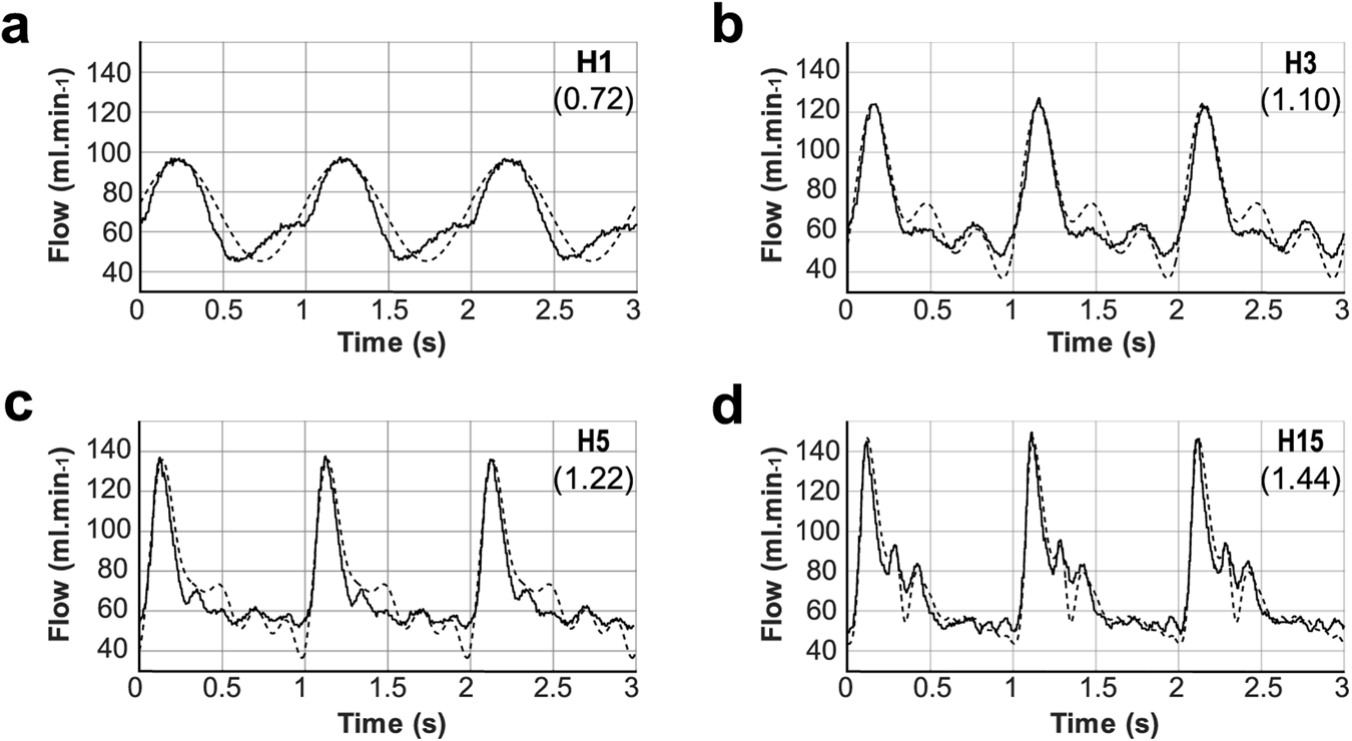
Flow patterns applied to the aneurysm. Four different target flow patterns were considered for the system (dashed lines). These included the first harmonic (**a**), the 1^st^ to 3^rd^ harmonics (**b**), the 1^st^ to 5^th^ harmonics (**c**), and the 1^st^ to 15^th^ harmonics (**d**) from the reference carotid flow, namely H1, H3, H5, and H15 respectively. The resultant effective experimental flow rate for each pattern is shown as a solid line. The pulsatility index of the measured flow is indicated in brackets.

Four flow patterns –H1, H3, H5, and H15 (Fig. 1)– were applied as input flow to a silicone model of a cerebral aneurysm of 10 mm diameter and 1.1 aspect ratio, which was therefore considered a large aneurysm. A carotid artery mean flow, the details of which have been published previously, was decomposed into harmonics^9,20^. The flow patterns H1, H3, and H5 were modelled by summing the 1st, 3rd, and 5th harmonics of the reference flow. Information on the flow complexity was gathered over the first 15 harmonics and the H15 flow pattern showed less than 1% variation from the original flow pattern^20^. The pulsatile flow complexity of the H1 to H15 patterns increased with the number of harmonics included in the signal, so their average values were normalised to the average flow pattern of the reference patient. Due to limitations of the regulation system, the flows generated by the test bench differed slightly from the input pattern (Fig. 1). Although the minimal flow rate was similar between these patterns (47 to 52 ml.min^−1^; Fig. 1), the peak flow rate increased from the H1 to the H15 patterns (97 to 146 ml.min^−1^; Fig. 1); consequently, the measured pulsatility indexes for H1, H3, H5, and H15 reached 0.72, 1.1, 1.22, and 1.44, respectively (Fig. 1). Increasing the number of harmonics included in the input signal also increased the number of peaks during one cardiac cycle, sharpening these peaks. The observed maximal acceleration of the flow rate ranged from 350 ml.min^−2^ to 1500 ml.min^−2^ for H1 to H15, respectively.

### Flow diverter physical properties

The flow diverters were designed for 3.5 mm, 4 mm, and 4.5 mm diameter arteries and produced with a 130° or 140° weave angle (Table 1). Because the target vessel diameter was 3.5 mm in all experiments, the porosity and pore density of the positioned flow diverters ranged from 44.2% to 68% and from 58.9 to 27.8 holes.mm^−2^, the number of holes being inversely proportional to the porosity (Table 1). Since the 140° weave angle configuration was associated with reduced flow diverter porosity compared to the 130° weave and smaller diameters with increased porosity, the lowest porosity was achieved with the 3.5 mm 140° flow diverter and the highest porosity with the 4.5 mm 130° configuration (Table 1).

**Table 1:** Flow diverter characteristics.

FD label	Diameter (mm) *relaxed*	Wire angle (°) *relaxed*	Porosity (%) *3.5 mm artery*	Pore density (holes/mm^2^) *3.5 mm artery*
S35a	3.5	140	44.2	58.9
S35b	3.5	130	53.1	45.98
S40a	4	140	61.1	45.1
S40b	4	130	63.3	35.2
S45a	4.5	140	66.9	35.63
S45b	4.5	130	68	27.81

### Experimental setup

**Figure 2:**
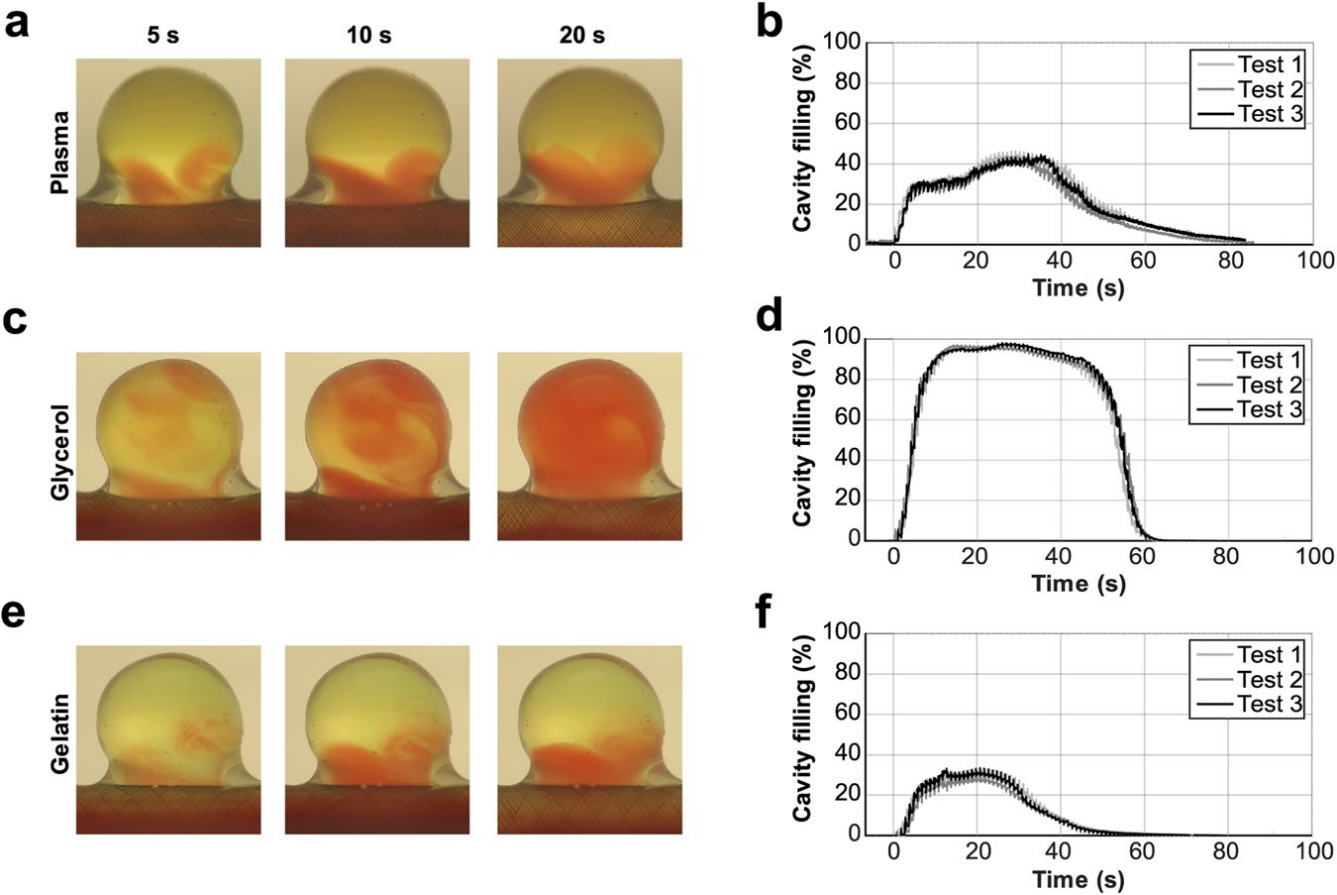
Selection of medium for the flow diverter assays. The use of human plasma (0.00172 Pa.s; panels **a, b**) as a medium for red blood cells was compared to glycerol dilution (0.00172 Pa.s; panels **c,d**), and gelatine solution (0.00207 Pa.s; panels **e,f**) in tests using the S40b flow diverter. Representative images of the aneurysm cavity at 5 s, 10 s, and 20 s are shown (**a,c,e**). The filling of the cavity is plotted over the time course of the experiments (**b,d,f**; n = 3).

Because reconstitution of blood requires strict compatibility of RBCs and human plasma batches, the number of *in vitro* experiments planned in this study was not feasible with such compounds. We therefore looked for a human plasma substitute for the *in vitro* assays. Two media were evaluated, namely isotonic glycerol solution with the viscosity of human plasma, and a commercially available gelatine solution (Geloplasma). The former is commonly used for *in vitro* experiments, whereas the latter is the main plasma substitute in clinical practice^21,22^. The S40b flow diverter was placed into the silicone aneurysm model and RBCs were injected into the circuit containing the fluid media. The distribution of the RBCs in the cavity was compared for human plasma, glycerol dilution, and gelatine solution (Fig. 2). The flow diverter position in the silicone model was not altered in the different experiments. Because the technical replicates (n = 3) were indistinguishable from each other, any difference between tests could only be due to the medium. None of the replacement media exactly matched the behaviour of the human plasma, perhaps because the gelatine solution has different viscosity (2.07 × 10^−3^ Pa.s 20 °C) from human plasma (1.72 × 10^−3^ Pa.s 20 °C) and both media had different densities (both 1014 kg.m^−3^) compared to human plasma (1040 kg.m^−3^). Although the glycerol solution had the same viscosity as the human plasma, RBC distribution inside the aneurysm with the gelatine solution was more similar to that with the plasma control (Fig. 2a,c,e). Likewise, the RBC distribution profile over time with the gelatine solution was closer to the plasma control than with the glycerol solution (Fig. 2b,d,f). Based on these conclusions, the subsequent experiments were performed using the gelatine solution as the human plasma substitute.

### Impact of the flow complexity on the flow diverter performance

**Figure 3:**
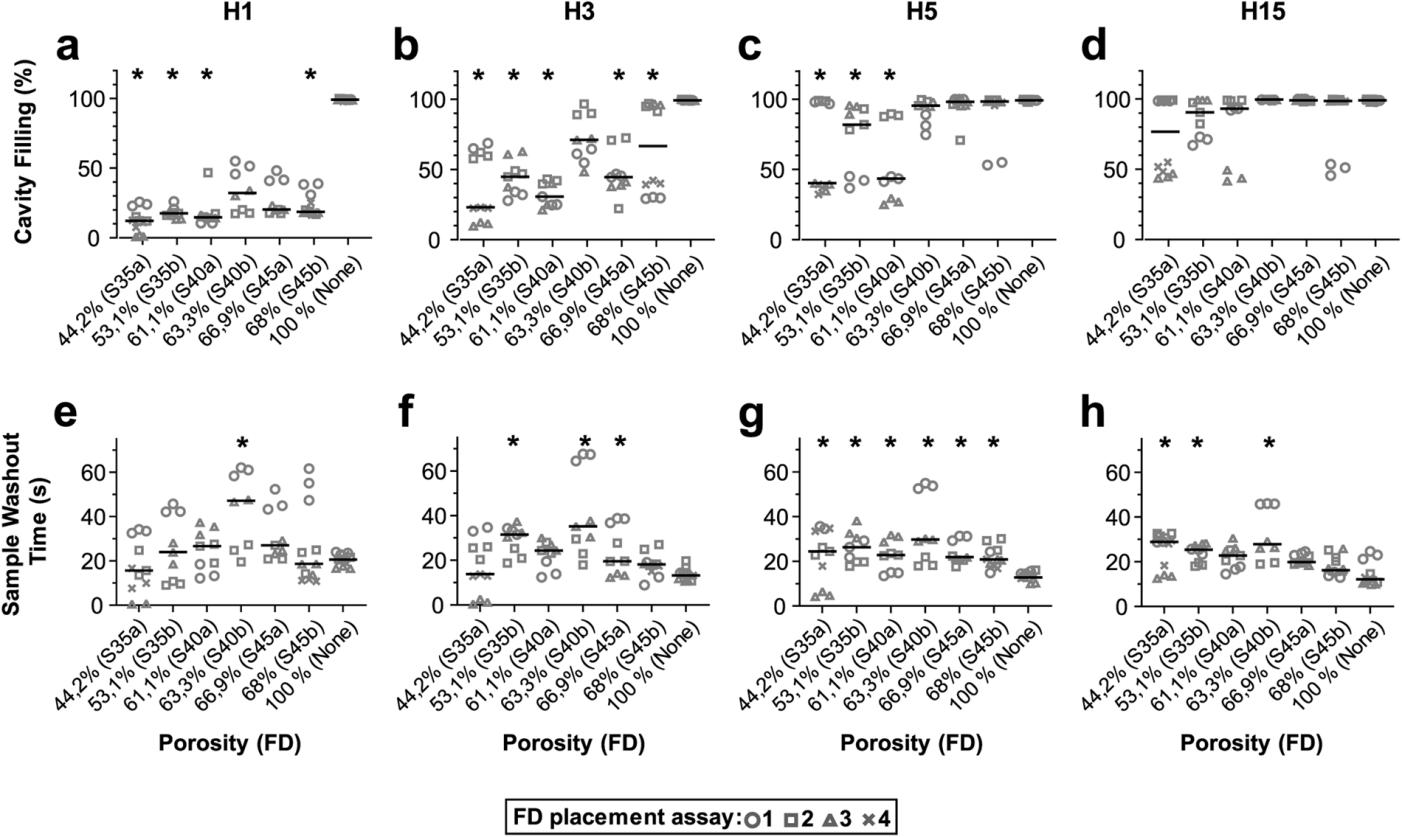
Impact of flow diverter placement on filling and washout of the aneurysm cavity by the red dye solution. Maximum cavity filling (upper panels) and sample washout time (lower panels) are plotted according to the flow diverter porosity as indicated. The corresponding flow diverter type is indicated in brackets. Each flow diverter was tested in 3 (S35b to S45a) or 4 (S35a, S45b) placements and each placement was evaluated in triplicate using flow patterns H1 (**a,e**), H3 (**b,f**), H5 (**c,g**), and H15 (**d,h**). The global median of the 3 or 4 flow diverter placement tests is shown as a solid line. Statistical difference compared to a 100% porosity condition was assayed using a Kruskal-Wallis post hoc Dunn’s test (*p < 0.05).

**Figure 4:**
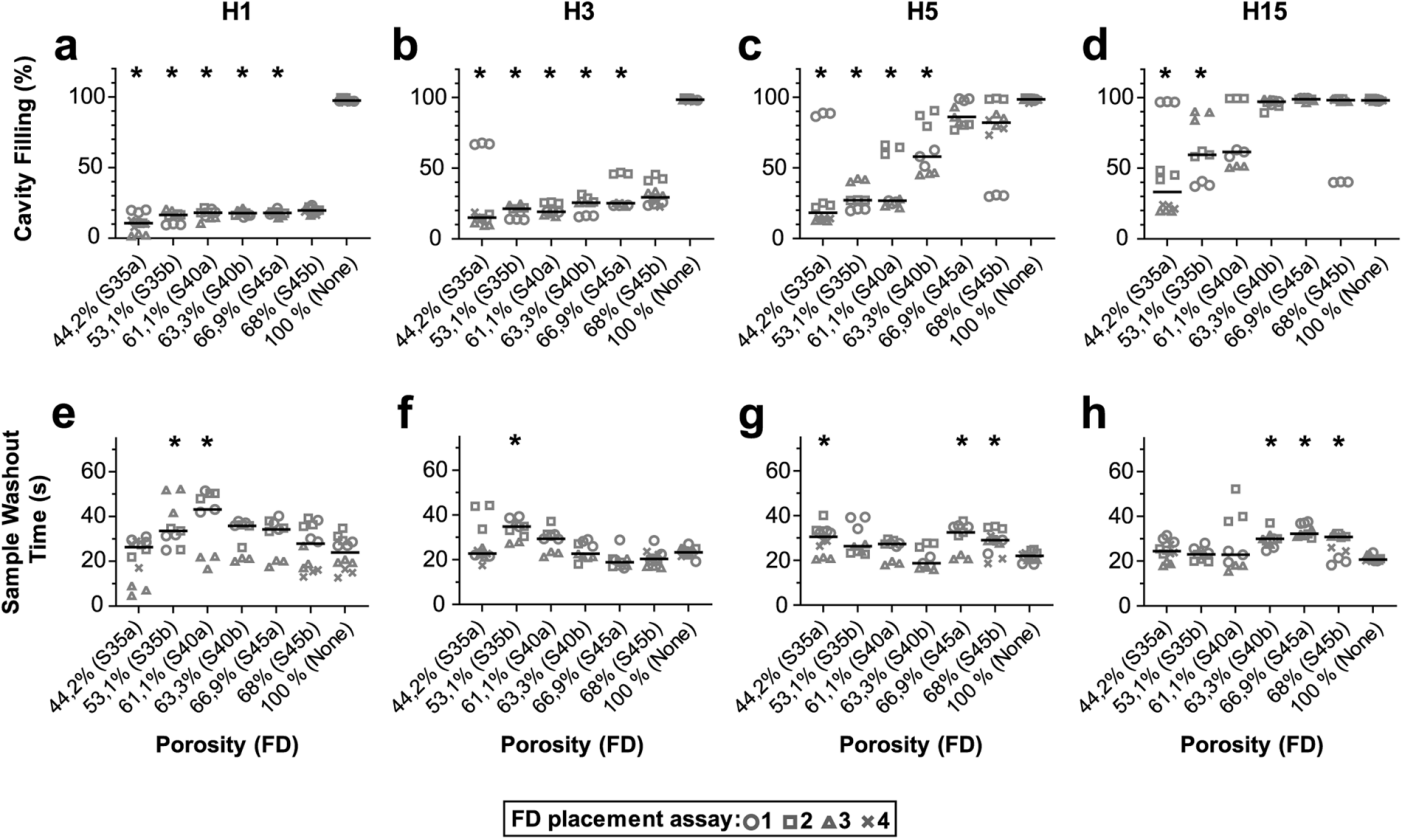
Impact of flow diverter placement on filling and washout of the aneurysm cavity by RBCs. Maximum cavity filling (upper panels) and sample washout time (lower panels) are plotted according to the FD porosity as indicated. The corresponding FD type is indicated in brackets. Each FD was assessed in 3 (S35b to S45a) or 4 (S35a, S45b) placements and each placement was assessed in triplicate using flow patterns H1 (**a,e**), H3 (**b,f**), H5 (**c,g**), and H15 (**d,h**). The global median of the3 or 4 flow diverter placement assessments is shown as a solid line. Statistical difference compared to a 100% porosity condition was tested using a Kruskal-Wallis post hoc Dunn’s test (*p < 0.05).

**Figure 5:**
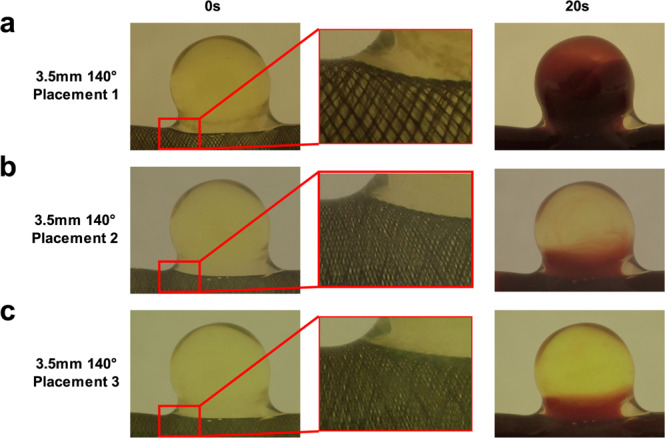
Effect of placement on mesh density of the flow diverter. Representative images of the aneurysm cavity at time 0 and 20 s during the 1^st^ (**a**), 2^nd^ (**b**), and 3^rd^ (**c**) placements of the S35a flow diverter (porosity 44.2%) are shown. The middle panels are enlargements of the 1eft panels.

Experiments were performed with the 6 flow diverter types, using red dye and RBCs to mimic the liquid and cellular fractions of the blood respectively. The flow patterns H1, H3, H5, and H15 were applied to the silicone model and videos of the dye/RBC entrance into and washout from the aneurysm cavity were post-processed to assess the maximum filling rate of the cavity and the mean washout time of the sample (Figs. 3 and 4). Control experiments performed without a flow diverter revealed that while the four flow patterns were able to completely fill the cavity, the number of harmonics present had a strong effect on the washout of the aneurysm cavity. The H15 pattern was associated with 38% and 23% shorter washout times than the H1 pattern using red dye and RBCs, respectively (Figs. 3, 4 and Suppl. Video 1, upper panels). The apparent velocity of the RBCs inside the cavity increased with the number of harmonics (Suppl. Video 1, upper panels). Similar differences between the four flow patterns were also observed when a flow diverter was placed at the cavity entrance (Suppl. Video 1, lower panels). To evaluate the impact of the harmonic complexity on the flow diverter’s ability to block the entrance of fluid into the aneurysm, the maximum filling of the aneurysm cavity and the mean washout time of the sample inside the cavity were compared with and without a flow diverter (Suppl. Video 1, Figs. 3 and 4). Although there was good reproducibility between technical replicates (n = 3) within one flow diverter type/flow pattern experiment, repeating the placement procedure led to clear variability between experiments (Figs. 3, 4 and Suppl. Video 2). The weave pattern also differed considerably across different placements of one flow diverter, although the same trained operator performed this step (Fig. 5).

### Effect of the flow diverter placement on the red dye flow

Using red dye, which mimicked plasma behaviour, most of the flow diverters were able to prevent dye entrance using flow patterns that had less than 5 harmonics (Fig. 3a,b). By contrast, all failed to show significant differences from control using the H15 pattern (Fig. 3d). As flow complexity increased from H3 to H5, lower porosity flow diverters were associated with greater flow cut-offs (Fig. 3b,c). The 140° flow diverter configuration systematically produced lower median values of cavity filling than did the 130° configuration (Fig. 3a–d). The lower cavity filling was associated with an increase in the washout time compared to control without flow diverter (Fig. 3). No apparent correlation could be observed between washout time and flow diverter porosity, but the 130° flow diverter configuration gave higher values than the same diameter 140° flow diverter, which had a lower porosity (Fig. 3e–h).

### Effect of the flow diverter placement on the RBC flow

The effect of the flow diverter on aneurysm flow was greater for RBCs than for red dye. Using the less complex flow patterns, namely H1 and H3, all flow diverters noticeably prevented RBC entrance into the aneurysm, all median values being less than 50% (Fig. 4a,b). Using more complex patterns, only the lower porosity flow diverters triggered an effective flow cut-off. While a porosity of less than 65% significantly prevented RBC entrance using the H5 pattern, it had to decrease to at least 55% for the H15 pattern, corresponding to a 7% and 30% increase in peak flow and maximum flow acceleration, respectively (Figs. 1 and 4c,d). Globally, for each placement assessment, the cavity filling values of the RBC experiments were higher than those using the red dye, suggesting a less efficient cut-off of the flow diverter for smaller particles (Figs. 3a–d and 4a–d). The geometry of the flow diverter also affected the maximum RBC cavity filling. Using the H1 and H3 flows, the RBC washout time was only altered for two flow diverters, namely S35b and S40a (Fig. 4e,f). With more complex flow patterns, only higher porosity flow diverters showed a significant increase in the RBC washout time, which could be up to 47% longer than control (Fig. 4g,h). Although they had different porosities, the 130° and 140° flow diverter configurations had similar median values of cavity filling (see S35b/S40a and S45a/S45b lanes, Fig. 4g,h). As for the red dye experiments, there was no correlation between porosity and flow diverter effect on washout time.

## Discussion

Mechanisms underlying thrombus formation inside aneurysms are still not well understood. However multiple possible contributing factors have been suggested, including flow reduction or redirection and reduced shear stress^5,23,24^. Many studies underline the importance of stagnation and slow flow inside the cavity^5,15,20^. Although promising, measuring direct thrombus formation inside an *in vitro* model does not reflect the reality of the long-term process, nor do CFD models^7,15^. Here, we evaluated the impact of flow diverters on flow inside a silicone aneurysm model when the complexity of the input pulsatile flow was restricted. This study focused on the ability of the injected sample to fill the cavity and the duration of its washout, mimicking the results obtained with digital subtractive angiography *in vivo*^25^.

The optical technique used here was based on the imaging of coloured markers, namely red dye (1 nm diameter) and RBCs (6–8 µm diameter)^26^. Because it is technically difficult to detect markers inside full blood due to its opacity, we tracked markers in the transparent liquid part of blood. The ideal medium would have been plasma. However, as this requires full compatibility with RBCs and as properties vary across donors, it would have been difficult to standardise for repeated experiments. Our results indicated that a gelatine solution, which is widely used as a plasma expander in the intensive care unit, was a better medium for the *in vitro* experiments than glycerol because it mimicked better the behaviour of RBCs in plasma. Although the glycerol solution matched plasma viscosity, it is a homogenous solution and therefore does not reflect the colloidal behaviour of plasma, by contrast to gelatine solution. Despite this limitation, glycerol is widely used in *in vitro* experiments due to its low cost, transparency and compatibility with RBCs^21,22^. Based on our results, the outcomes of such experiments, as well as CFD simulations restricted to the liquid phase only, should be interpreted with care.

Current methods to study the flow diverter impact over aneurysms included *in vivo* experimentation in animals^16,18^, *in vitro* experiments using silicone models^15,22,26^ and CFD simulations^7,11,13,20^. *In vivo* approaches give physiological information on the flow diverter effect such as blood flow alteration, vessel geometry or clot formation in long time range^16,18,25^. However, this strategy is strongly limited by ethical concerns, an access to suited animal facilities and the associated experimental constraints. In addition, information about the flow inside the aneurysm and the RBCs interaction with the flow diverter can hardly be retrieved^25^. The CFD simulations can overcome these limitations and deliver physiological information anywhere in the model such as velocity, viscosity or haematocrit level^7,11,13,20^. However, the success of the CFD simulation depends on the state-of-art of the mathematical model and its associated computational cost to solve it, which are the current limitations for whole blood simulation^27,28^. Moreover, the results are interpretive and need to be validated using *in vivo* or experimental data^28^. These experiments evaluate the physical quantities of a biological process but are limited by the available technology to perform the observations. As the use of whole blood is challenging, most experiments focused on the liquid part of the blood using glycerol or blood mimicking fluid, including the particle image velocimetry where particles are used as a fluid tracker^15,22^. Our experimental strategy included the study of the RBCs interacting with the flow diverter in an aneurysm and enlightened their role in the flow diverter treatment. While the present experimental setup focused on RBC batch behaviour, further developments focus on single cell tracking within whole blood using fluorescent labelling.

The blood flow pattern varies within the cardiovascular system due to the geometry and physical properties of arteries, among other parameters^11,29^. In the cerebral tree, flow is dampened between the carotid arteries and the arterioles where flow becomes a simple waveform^11^. Moreover, these properties change with aging. Because vessels become more rigid with age, flow complexity is increased along the cerebral tree in the elderly^30^. Many studies have reported that flow diverter outcomes depend on the flow velocity^8,13,14^. It has also been shown that the impact of the flow diverter is less during the systolic phase than during the diastolic phase and that this is related to the blood flow velocity only^14^. This finding suggests that the complexity of the flow pattern is crucial and is likely dependent on the peak sharpness, which reflects the maximum velocity, and the flow acceleration/deceleration. To further investigate this issue, we separately tracked the behaviour of liquid and particles in the aneurysm model. Using four flow patterns with increasing harmonic complexity and pulsatility indexes, we observed that the flow-diverter-associated flow cut-off decreased with increasing pulsatility indexes. In our data, although the maximum flow value was only increased 1.5-fold, we observed a 5-fold increase in the maximum acceleration value between the H1 and H15 profiles. The increased acceleration or the sharpness of the peak better reflected the lower cutting of the flow by some flow diverters. As previously described, the impact of the pulsatility index on flow diverter cut-off was also porosity dependent^4,21^. The flow diverters with porosity less than 65% reduced the flow for pulsatile indexes of up to 1.4, whereas all flow diverters successfully cut flow when the pulsatile index was less than 1. In a previous study, we observed that only one third of patients showed a cerebral pulsatile index greater than 1, and none of them reached the 1.4 value^31^. However, pulsatile indexes greater than 1.4 have been reported for external carotid arteries and middle meningeal arteries in healthy volunteers and for internal carotid arteries and middle cerebral arteries in patients with hypertension or diabetes^12,32,33^. Although the washout time of the sample was mildly affected by placement of a flow diverter, a large area of the aneurysm remained was not reached by the marker following use of lower porosity flow diverters, whichever flow pattern was used. Such zones have been shown to be prone to thrombus formation^7,15,20^. Of note, these threshold pulsatile indexes may be lower using MRI measurements, because pulsatile indexes calculated from Doppler analysis are higher than those from MRI, due to underestimation of the systolic peak^34,35^.

The flow diverters had a different effect on the flow behaviour of the liquid fraction of blood, which was imaged using red dye, and on the RBCs, which mimicked the cellular components of blood. In the *in vitro* experiments, RBC distribution was affected more by flow diverters than was the distribution of the red dye. Many factors may be involved in this difference. These two markers have different light absorbances, altering the detection of their distribution within the silicone model. The effect of gravity on the RBCs may also play a role^26^. The difference may also be related to the size of the red dye and RBC particles and to the higher energy dissipation of the RBCs on the flow diverter wires because of their larger size. Nonetheless, the difference suggests that CFD simulations that rely on fluids alone may underestimate the effect of flow diverters on the flow inside the aneurysm cavity, especially since the key players in thrombus formation are most likely soluble or small size particles, namely the platelets^7,20^. Because of technical limitations, experiments using whole blood and tracking plasma molecules or platelets are not feasible but would provide better understanding of the effects of flow diverters on thrombus formation.

The highest flow cut-offs were achieved when the target diameter of the flow diverter corresponded to the vessel diameter because oversizing led to increased porosity^36^. Porosity values did not correlate with the cavity filling results, nor did the pore density in the red dye experiments. There was a discrepancy between the results obtained for the 130° and the 140° angle wire flow diverters, the latter being more effective at preventing aneurysm filling by the dye. These results are in accordance with previous work showing that flow diverter performance cannot be solely described using porosity^22,37^. Flow diverter performance depends on weave pattern, wire diameter, geometry of targeted vessel(s) and on flow properties^13,22,36–38^. In addition to these parameters, we observed an unexpected variability in our experiments from day to day, although technical reproducibility within one day was high. This variability was linked to placement of the flow diverter, which led to differences in the porosity along the length of the flow diverter *in situ*. Together, these results indicate that the flow diverter placement may be more important for flow diverter efficacy than any other parameter. This is in accordance with previous studies showing that in *in vivo* experiments, the predicted 72% porosity of a flow diverter can vary from 17% to 77% following placement, and this was associated with thrombosis outcome^18,39^. Slight differences in placement led to drastically different flow trajectories inside the aneurysm^40,41^. As a consequence, a jet flow was observed in some cases, triggering a local increase in the shear stress on the aneurysm wall, which was associated with an increased risk of aneurysm rupture^5^. In our study, when the same operator placed the same flow diverter in a conserved aneurysm geometry, the variability in the extent of cavity filling ranged from 1.7–77%. This suggests that during surgery with different aneurysm/vessel configurations, the physician is unlikely to place the flow diverter as intended. Better tools should be developed to help the physician in this procedure, for example, mechanical or computer-assisted markers^3,5^.

This study has several limitations. Firstly, only one aneurysm shape, one flow diverter design, and one operator for flow diverter placement were used, restricting the relevance of our results for other conditions but giving good reproducibility. Secondly, because of the flexible material used, the model imitated the elasticity of the vessel, although the elasticity and surface properties remain different from those of actual vessels and this difference may have influenced the flow diverter outcomes. Thirdly, the post-processing image algorithm, which detects the markers was based on the colour range. The analysis could have been performed using the intensity of the colour and placed under ideal light conditions using a dark room with controllable artificial light conditions. However, our preliminary experiments revealed that these parameters accounted for less than 5% of error. Fourthly, all acquisitions were performed using an HD camera which produced only a 2D side view projection of the aneurysm. Future experiments could use a 3D analysis of the flow, using either a second HD camera placed on a different plane or a CT-scanner. Finally, we focused on a characteristic reference flow to generate the four flow patterns used in this study^20^. However, because the pulsatile index does not reflect the full complexity of the signal, different pulsatile flow patterns with the same pulsatile index may have been evaluated. To better unravel the importance of flow complexity of the flow diverter, these experiments should be repeated with conserved pulsatile indexes but different flow patterns showing different peak profiles.

Together, the present data highlight the importance of the measurement of flow complexity, herein described using the pulsatile index, for the correct selection of a flow diverter for treatment of an aneurysm. Our results also reveal the impact of flow diverter placement per se on the modification of flow inside the aneurysm, probably as a result of altered physical properties of the flow diverter *in situ*. Progress in flow diverter positioning is eagerly awaited to overcome this crucial problem.

## Methods

### Sample collection

RBC solution (77%) and human plasma (1.72 × 10^−3^ Pa.s viscosity, 1040 kg.m^−3^ density at 20 °C) were provided by La transfusion du Sang ASBL (Charleroi, Belgium). Red dye solution was prepared from Ponceau 4 R powder diluted into the medium used in the experiment (1 mg.ml^−1^). Glycerol solution was diluted to reach 1.72 × 10^−3^ Pa.s viscosity (1014 kg.m^−3^ density at 20 °C) and equilibrated to isotonicity with NaCl. Geloplasma (Fresenius Kabi SA) was used as the gelatine solution (2.07 × 10^−3^ Pa.s viscosity, 1014 kg.m^−3^ density at 20 °C).

### *In vitro* test bench settings

The full procedure has been described in previous work by Chodzyński *et al*.^26^. Briefly, the experiments were carried out using the spherical transparent silicone model placed in the *in vitro* test bench developed previously^19^. The test bench was set at 37 °C. The pulsatile flow patterns were generated using the Fourier series applied to a previously published pulsatile flow pattern from the carotid artery to impose different harmonics as follows^20^:

$${\rm{Q}}({\rm{t}})=\frac{{\rm{a}}0}{2}+{\sum }_{{\rm{n}}=1}^{{\rm{N}}}[{{\rm{a}}}_{{\rm{n}}}\,\sin ({\rm{nt}})+{{\rm{b}}}_{{\rm{n}}}\,\cos ({\rm{nt}})]\,{\rm{for\; N}}\ge 0$$

where n = [1, 3, 5, 15] are the harmonics used to produce the input pulsatile flow, namely H1, H3, H5, and H15 respectively (Fig. 1, dashed lines). The pulsatility index was calculated as follow (Fig. 1):

$${\bf{PI}}=({\rm{peak}}\,{\rm{systolic}}\,{\rm{velocity}}\,-\,{\rm{end}}\,{\rm{diastolic}}\,{\rm{velocity}})/{\rm{time}}\,{\rm{averaged}}\,{\rm{velocity}}$$

The heartbeat rate was kept at 60 Hz. The flow diverters used in this study were multilayer braiding mesh structures 20 mm in length (Multilayer Flow Modulator, Cardiatis SA). Experiments were carried out on the 6 FD configurations: 3 diameters (3.5, 4, and 4.5 mm) and 2 braid angles (130° and 140°), resulting in different porosities (Table 1).

### *In vitro* experimental procedure

The experimental procedure was similar to that used in previous work^26^. Briefly, RBCs (1 ml) or red dye solution (1 ml) were injected upstream of the aneurysm at time 0 for 17 s. Aneurysm orientation was upwards. Experimental replicates (n=3) of each flow pattern corresponding to a single flow diverter placement were performed the same day with dye and RBCs. Each flow diverter placement was repeated in 3 independent series of experiments for S35a and S45b and 4 for S35b to S45a. Control experiments without the flow diverter were performed for each placement and flow pattern.

### Image processing

The procedure was identical to that used in previous work^26^. Briefly, images were recorded using a full HD camera (Panasonic 700 SD, 1080p) at 50 fps. Processing was performed using Matlab 2015 (MathWorks). The analysis steps were conducted as previously reported except that the colour threshold in the HSV colour map was 330° to 30° [ref. ^26^]. The quantity of RBCs/dye passing through the aneurysm was assessed using the following equation describing the Normalised Mean Filling Fraction (NMFF) defined as follows:

$$NMFF=\frac{1}{T}{\int }_{0}^{T}\frac{N(t)}{{N}_{0}}dt$$

where: *N*_0_ is the number of white pixels assuming complete filling of the aneurysm by RBCs, *N*(t) is the number of white pixels covering the aneurysm surface, $\frac{N(t)}{{N}_{0}}\le 1$ is the fraction of white pixels per time, and *T* is the full experiment duration, equal for all experiments. During all experiments, T was set as 100 s. Maximal cavity filling was defined as the maximum percentage of $\frac{N(t)}{{N}_{0}}$ during the time course of the experiment. The mean sample washout time was calculated as follows: the area under the curve $\frac{N(t)}{{N}_{0}}$ from 17 s following first detection of red dye or RBCs in the cavity until 100 s was divided by the maximum value of the curve.

### Statistical analyses

Flow diverter placement results were compared to controls using a Kruskal-Wallis post hoc Dunn’s test using p < 0.05 to indicate significant differences. Statistical analyses were performed on the flow diverter placement replicates using the mean values of the technical replicates performed on the same day.

## Data Availability

The datasets analysed during the current study are available from the corresponding author on reasonable request.

## Supplemenatry information

Supplemenatry information.
Supplemenatry information 2.
Supplemenatry information 3.

## References

[1] Steiner T (2013). European Stroke Organization guidelines for the management of intracranial aneurysms and subarachnoid haemorrhage. Cerebrovasc. Dis..

[2] Chowdhury T, Cappellani RB, Sandu N, Schaller B, Daya J (2013). Perioperative variables contributing to the rupture of intracranial aneurysm: an update. Sci. World J..

[3] Pierot L (2011). Flow diverter stents in the treatment of intracranial aneurysms: Where are we?. J. Neuroradiol..

[4] Kim SR (2008). Anatomic results and complications of stent-assisted coil embolization of intracranial aneurysms. Interv. Neuroradiol..

[5] Etminan N, Rinkel GJ (2016). Unruptured intracranial aneurysms: development, rupture and preventive management. Nat. Rev. Neurol..

[6] Larrabide I (2013). Intra-Aneurysmal Pressure and Flow Changes Induced by Flow Diverters: Relation to Aneurysm Size and Shape. AJNR Am. J. Neuroradiol..

[7] Peach TW, Ngoepe M, Spranger K, Zajarias-Fainsod D, Ventikos Y (2014). Personalizing flow-diverter intervention for cerebral aneurysms: from computational hemodynamics to biochemical modeling: personalizing flow-diverter intervention for cerebral aneurysms. Int. J. Numer. Meth. Biomed. Engng..

[8] Morales HG (2016). Does Arterial Flow Rate Affect the Assessment of Flow-Diverter Stent Performance?. AJNR Am. J. Neuroradiol..

[9] Wright G, Furness A (1985). What Is Pulsatile Flow?. Ann. Thorac. Surg..

[10] de Riva N (2012). Transcranial Doppler Pulsatility Index: What it is and What it Isn’t. Neurocrit. Care.

[11] Grinberg L, Anor T, Cheever E, Madsen JR, Karniadakis GE (2009). Simulation of the human intracranial arterial tree. Philos. Trans. Royal Soc. A.

[12] Zarrinkoob L (2016). Aging alters the dampening of pulsatile blood flow in cerebral arteries. J. Cereb. Blood Flow Metab..

[13] Mut F (2014). Effects of changing physiologic conditions on the *in vivo* quantification of hemodynamic variables in cerebral aneurysms treated with flow diverting devices: effects of changing physiologic conditions on aneurysm hemodynamics. Int. J. Numer. Meth. Biomed. Engng..

[14] Larrabide I, Geers AJ, Morales HG, Bijlenga P, Rüfenacht DA (2016). Change in aneurysmal flow pulsatility after flow diverter treatment. Comput. Med. Imaging Graph..

[15] Gester K (2016). *In Vitro* Evaluation of Intra-Aneurysmal, Flow-Diverter-Induced Thrombus Formation: A Feasibility Study. AJNR Am. J. Neuroradiol..

[16] Sadasivan C, Cesar L, Seong J, Wakhloo AK, Lieber BB (2009). Treatment of Rabbit Elastase-Induced Aneurysm Models by Flow Diverters: Development of Quantifiable Indexes of Device Performance Using Digital Subtraction Angiography. IEEE Trans. Med. Imaging.

[17] UCAS Japan I (2012). The natural course of unruptured cerebral aneurysms in a Japanese cohort. N. Engl. J. Med..

[18] Darsaut TE, Bing F, Salazkin I, Gevry G, Raymond J (2011). Testing Flow Diverters in Giant Fusiform Aneurysms: A New Experimental Model Can Show Leaks Responsible for Failures. AJNR Am. J. Neuroradiol..

[19] Chodzyński KJ (2015). An *in vitro* test bench reproducing coronary blood flow signals. Biomed. Eng. Online.

[20] Malaspinas O (2016). A spatio-temporal model for spontaneous thrombus formation in cerebral aneurysms. J. Theor. Biol..

[21] Lieber BB, Stancampiano AP, Wakhloo AK (1997). Alteration of hemodynamics in aneurysm models by stenting: Influence of stent porosity. Ann. Biomed. Eng..

[22] Lieber BB, Livescu V, Hopkins LN, Wakhloo AK (2002). Particle Image Velocimetry Assessment of Stent Design Influence on Intra-Aneurysmal Flow. Ann. Biomed. Eng..

[23] Cohen JE (2007). Spontaneous thrombosis of cerebral aneurysms presenting with ischemic stroke. J. Neurol. Sci..

[24] Whittle IR, Dorsch NW, Besser M (1982). Spontaneous thrombosis in giant intracranial aneurysms. J. Neurol. Neurosurg. Psychiatry.

[25] Pereira VM (2013). A DSA-Based Method Using Contrast-Motion Estimation for the Assessment of the Intra-Aneurysmal Flow Changes Induced by Flow-Diverter Stents. AJNR Am. J. Neuroradiol..

[26] Chodzyński KJ (2016). Does the gravity orientation of saccular aneurysms influence hemodynamics? An experimental study with and without flow diverter stent. J. Biomech..

[27] Czaja, B., Závodszky, G., Azizi Tarksalooyeh, V. & Hoekstra, A. G. Cell-resolved blood flow simulations of saccular aneurysms: effects of pulsatility and aspect ratio. *J. R. Soc. Interface* **15** (2018). 10.1098/rsif.2018.0485 PMC6170784 30257923

[28] Dennis KD, Kallmes DF, Dragomir-Daescu D (2017). Cerebral aneurysm blood flow simulations are sensitive to basic solver settings. J. Biomech..

[29] Masuda M (2013). Evaluation of blood flow velocity waveform in common carotid artery using multi-branched arterial segment model of human arteries. Biomed. Signal Proces. Control.

[30] Azhim, A. *et al*. Exercise Improved Age-associated Changes in the Carotid Blood Velocity Waveforms. *JBPE* **10** (2007).

[31] Eker OF (2015). MR Derived Volumetric Flow Rate Waveforms of Internal Carotid Artery in Patients Treated for Unruptured Intracranial Aneurysms by Flow Diversion Technique. J. Cereb. Blood Flow Metab..

[32] Harris S, Reyhan T, Ramli Y, Prihartono J, Kurniawan M (2018). Middle Cerebral Artery Pulsatility Index as Predictor of Cognitive Impairment in Hypertensive Patients. Front. Neurol..

[33] Lee KY, Sohn YH, Baik JS, Kim GW, Kim J-S (2000). Arterial Pulsatility as an Index of Cerebral Microangiopathy in Diabetes. Stroke.

[34] Balédent O (2006). Brain hydrodynamics study by phase-contrast magnetic resonance imaging and transcranial color doppler. J. Magn. Reson. Imaging.

[35] Sadat Safavi, T., Fatouraee, N. & Soleimani, E. Quantification of Blood Flow Velocity of the Internal Carotid Artery: A Comparison Between Phase-Contrast MRI and Doppler Ultrasound. In *2017 24th National and 2nd International Iranian Conference on Biomedical Engineering (ICBME)* 330–333, 10.1109/ICBME.2017.8430232 (2017).

[36] Mut F, Cebral JR (2012). Effects of Flow-Diverting Device Oversizing on Hemodynamics Alteration in Cerebral Aneurysms. AJNR Am. J. Neuroradiol..

[37] Kim YH, Xu X, Lee JS (2010). The Effect of Stent Porosity and Strut Shape on Saccular Aneurysm and its Numerical Analysis with Lattice Boltzmann Method. Ann. Biomed. Eng..

[38] Liou T-M, Liou S-N, Chu K-L (2004). Intra-Aneurysmal Flow With Helix and Mesh Stent Placement Across Side-Wall Aneurysm Pore of a Straight Parent Vessel. J. Biomech. Eng..

[39] Darsaut TE, Bing F, Salazkin I, Gevry G, Raymond J (2012). Flow diverters failing to occlude experimental bifurcation or curved sidewall aneurysms: an *in vivo* study in canines. JNS.

[40] Hirabayashi M, Ohta M, Rüfenacht DA, Chopard B (2003). Characterization of flow reduction properties in an aneurysm due to a stent. Phys. Rev. E.

[41] Makoyeva A, Bing F, Darsaut TE, Salazkin I, Raymond J (2013). The Varying Porosity of Braided Self-Expanding Stents and Flow Diverters: An Experimental Study. AJNR Am. J. Neuroradiol..

