# Supplementary material for: The impact of arterial flow complexity on flow diverter outcomes in aneurysms
Source: Sci Rep. 2020 Jun 25;10:10337. doi: 10.1038/s41598-020-67218-9 (PMC7316819; doi:10.1038/s41598-020-67218-9)
Supplement: Supplementary file 1 — Supplemenatry information. [file 41598_2020_67218_MOESM1_ESM.pdf]

The impact of arterial flow complexity on flow diverter outcomes in aneurysms

Kamil Chodzyǹski, Pierrick Uzureau, Vincent Nuyens, Alexandre Rousseau, Gregory Coussement, Karim Zouaoui Boudjeltia

Supplementary Video 1: Effect of flow diverter placement on cavity filling. Real time video of RBC injection into the aneurysm cavity without (upper panels) and with (lower panels) the S40a flow diverter using H1, H3, H5, and H15 flow patterns (representative assessment of placement 1).

Supplementary Video 2: Effect of flow diverter placement on cavity filling. Real time video of RBC injection into the aneurysm cavity following placement of the S35a FD using H15 flow patterns. Representative assessment of the 4 flow diverter placements (1-4).
